# Highly Reactive Thermite Energetic Materials: Preparation, Characterization, and Applications: A Review

**DOI:** 10.3390/molecules28062520

**Published:** 2023-03-09

**Authors:** Xiaogang Guo, Taotao Liang, Md. Labu Islam, Xinxin Chen, Zheng Wang

**Affiliations:** 1 Chongqing Key Laboratory of Inorganic Special Functional Materials, College of Chemistry and Chemical Engineering, Yangtze Normal University, Chongqing 408100, China; 2 Chongqing Sports Medicine Center, Southwest Hospital, The Third Military Medical University, Chongqing 400038, China; 3 School of Chemistry and Chemical Engineering, Beijing Institute of Technology, Beijing 102488, China

**Keywords:** energetic materials, nanostructures, thermites, systematic classification, exothermic performance, broad prospects

## Abstract

As a promising kind of functional material, highly reactive thermite energetic materials (tEMs) with outstanding reactive activation can release heat quickly at a high reaction rate after low-energy stimulation, which is widely used in sensors, triggers, mining, propellants, demolition, ordnance or weapons, and space technology. Thus, this review aims to provide a holistic view of the recent progress in the development of multifunctional highly reactive tEMs with controllable micro/nano-structures for various engineering applications via different fabricated techniques, including the mechanical mixing method, vapor deposition method, assembly method, sol-gel method, electrospinning method, and so on. The systematic classification of novel structured tEMs in terms of nano-structural superiority and exothermic performance are clarified, based on which, suggestions regarding possible future research directions are proposed. Their potential applications within these rapidly expanding areas are further highlighted. Notably, the prospects or challenges of current works, as well as possible innovative research ideas, are discussed in detail, providing further valuable guidelines for future study.

## 1. Introduction

Energetic materials (EMs), regarded as a kind of promising functional material, can generate enormous amounts of thermal energy at a high rate of heat release within a surprisingly short time (<1 s) after a small energy stimulation, and are widely used in the fields of sensors, trigger, mining, propellants, demolition, ordnance or weapons, civilian and military [1,2,3,4]. With the rapid development of nanotechnology, designing nano-energetic materials (nEMs) with nanostructured-fuel and oxidizer has attracted increasing attention, especially in the last two decades [5,6]. Compared with traditional micro-EMs, nEMs have a faster burning rate and a more intense loading process due to a larger contact area and shorter mass transfer distance among reactants during the exothermic reaction process [7,8], and their superior structure and performance advantages allow them to exhibit wider application potentials in micro-electro-mechanical systems (MEMS), lab-on-a-chip devices, miniaturized or sophisticated weapons and equipment [9,10,11,12,13,14].

As the promising subclass of nEMs, highly reactive thermite EMs (tEMs) with an outstanding heat release ability due to high reaction enthalpy have drawn increasing interest and have wide application prospects in the fields of sensors, microelectronics, propeller machinery, miniaturized detonating primers and advanced military industry [15]. Generally, the metal in tEMs acts as the high energy fuel, and the other reactants act as oxidizing agents, including metal or nonmetal (Ni [16], Ti [17], etc), oxidizer (R_x_O_y_) (R = Fe [18,19,20], Cu [21,22,23], Bi [24], Co [25,26], Ni [27], Mo [28], W [29], etc.), inorganic salt (polytetrafluoroethylene (PTFE) [30], KMnO_4_ [31], NaClO_4_ [32], etc.), organic molecule (trinitrotoluene (TNT) [33], Hexanitrohexazisowootane (CL-20) [34], trimethylenetrinitramine (RDX) [35] etc.), 3D organic framework [36], etc. For example, activated Al/Co_3_O_4_ tEM was prepared by high-energy ball milling, as reported by R. J. Yang group, and the reaction properties of the Al/Co_3_O_4_ powders with the water steam were closely related to the milling time [25]. K.S. Martirosyan et al. systematically studied eight energetic systems: nano-Al@Fe_2_O_3_, Al@Fe_3_O_4_, Al@MnO_2_, Al@MoO_2_, Al@WO_3_, Al@Bi_2_O_3_, and Al@CuO, demonstrating that the Al@Bi_2_O_3_ generated the highest peak pressure of ~10 MPa due to the lower boiling point of metal Bi, showing great application in gas generators [37]. Moreover, due to long-range electrostatic force and covalent interactions, carbon fiber oxidant (CFO) was introduced to design CFO/Al/Bi_2_O_3_, which can detonate RDX without primary explosives and bridge film, largely improving the reliability of the ignition device [38]. Notably, the nanostructures largely grant tEMs advantages in detonation performance and exothermic process and show wider potential applications in the fields of war industry, electronics, national defense, etc.

Designing tEMs with different novel microstructures and improving the energy-releasing capacity of tEMs have been two of the most essential research focuses, and the corresponding amount of important literature regarding tEMs is growing fast. In fact, some reviews are available for energetic materials [39,40,41]. For example, Y.M. Maximov summarized the common exothermic reactions and their main utilization of metallurgical applications, synthesis of materials, etc. [40]. The preparation methods and fundamental properties of the various kinds of core-shell structured nEMs were concluded, and the future related research challenges (e.g., not enough research on the combustion mechanisms of complex nEMs) was proposed by K.L. Zhang group [3]. However, a large quantity of reported emerging oxidizer materials have been explored to optimize the performances of tEMs, especially in the last few years. And the family system or classification of tEMs is extended accordingly. For example, in situ synthesis yielded novel metastable intermixed core-shell n-Al@annic acid@M(IO_3_)x (M = Fe, Cu, Bi) nanocomposites with Al as the core and annic acid as an interfacial layer, which demonstrated higher thermal reactivity, larger volume reaction heat, higher combustion efficiency, and a faster burning rate than mechanically mixed n-Al/M(IO_3_)x [42], showing a wide application. Moreover, several novel and high-efficiency synthetic technologies (e.g., two-step ball milling) are emerging in the latest reports. Thus, it is urgent to update research progress or review on tEMs.

**Figure 1:**
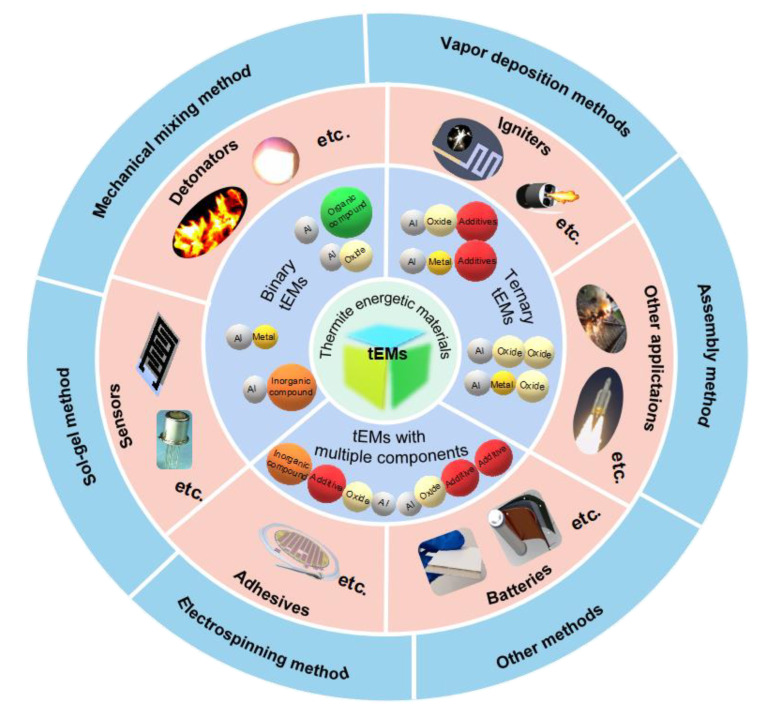
The classification, application and preparation process of highly reactive tEMs.

This review focuses on nEMs, mainly reviewing tEMs in terms of classification, fabrication technique and breadth of application (Figure 1). Section 2 summarizes the more complete classification of tEMs. Notably, the advantages and drawbacks of different preparation methods are clarified in Section 3. Moreover, we will also highlight the recent impressive application prospects of emerging tEMs and their associated challenges in Section 4. Finally, the conclusions are elaborated in Section 5.

## 2. Classification of Highly Reactive Thermite EMs (tEMs)

It cannot be denied that after domestic and foreign exploration in recent decades, the research of tEMs has been gradually systematized, theorized and diversified. In general, tEMs can be divided into binary and multivariate materials according to the type of components.

### 2.1. Binary tEMs

#### 2.1.1. Al/Metal Oxide tEMs

Regarding the oxidizer components of tEMs, metal oxides are the most frequently used due to their diversity, easy accessibility, and high thermal reactivity with Al, which is generally regarded as the most intensively researched energetic system. They can release energy quickly because of the classic aluminothermic reaction, which provides their own oxygen supply due to the presence of oxygen and can be self-sustaining. The commonly used metal oxides include Fe_2_O_3_, MnO_2_, Cr_2_O_3_, WO_3_, Bi_2_O_3_, CuO, NiO, etc. [43]. The microstructures of Al/metal oxide composite energetic materials are adjusted by different fabrication methods, and the choice of metal oxide is mainly determined according to different application requirements.

**Figure 2:**
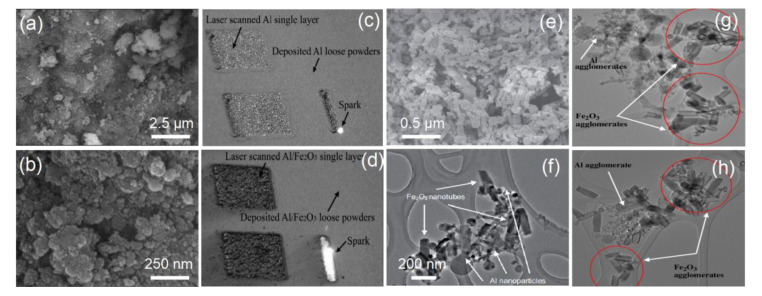
Field emission scanning electron microscopy (FESEM) image (**a**,**b**) of Al/Fe_2_O_3_ nEMs by soft template self-assembly with sol-gel process [46] Copyright 2015, Journal of Solid State Chemistry, a comparative visual observation of laser melting method process on a thick layer of (**c**) Al powder and (**d**) Al/5 wt% Fe_2_O_3_ powder mixture [47] Copyright 2012, Advanced Engineering Materials, the (**e**) FESEM and (**f**) transmission electron microscope (TEM) image of self-assembled Al/Fe_2_O_3_ nEMs, and followed by the (**g**) and (**h**) TEM images of physically solvent-mixed Fe_2_O_3_ nanotubes-Al nanoparticles sample [48] Copyright 2010, Combustion and Flame.

As a classical thermite system, Al/Fe_2_O_3_ EMs can release a lot of thermitic reaction heat under the excitation of small external energy. The heat-release (Q) of the theoretical stoichiometric Al/Fe_2_O_3_ is more than 3.9 kJ/g, and its adiabatic temperature is up to 3135 K [44]. Different fabrication techniques have been explored to obtain Al/Fe_2_O_3_ EMs, including mechanical mixing, self-assembly, electrophoretic deposition, etc. [3,18,20,45]. For example, Y.J. Luo group prepared Al/Fe_2_O_3_ EMs via a self-assembly method combined with a sol-gel process method, and the nano-Al particles did not aggregate and were coated by the nano-Fe_2_O_3_ particles, providing a high effective contact area between the fuel and oxidizing agent (Figure 2a,b). Moreover, the heat release of the assembly-Al/Fe_2_O_3_ sample can reach ~2.0 kJ/g [46]. The advanced isoconversional kinetic analysis of Fe_2_O_3_-2Al thermite reaction was studied by M.J.S. de Lemos group, which is used for plug and abandonment of oil wells [19]. The Al/Fe_2_O_3_ composite with promising three-dimensional structures (Figure 2c,d) was constructed by L Hao team using a selective laser melting method [47]. N.N. Thadhani et al. reported the Al/Fe_2_O_3_ EMs using nano-Al particles and the Fe_2_O_3_ nanotubes via facile mixed self-assembled technique, and the commercially purchased nano-Al powders are relatively evenly distributed around the Fe_2_O_3_ nanotubes (Figure 2e), largely contributing to the adequate thermite reaction. The rapid reaction propagation occurs throughout the self-assembled Al/Fe_2_O_3_ EMs when nano-Al starts to melt, and reactive sites quickly spread from the melting of the Al core to the surface of the Al particles to react with the surrounding oxidizers-Fe_2_O_3_ (Figure 2f) [48], and distinct segregated agglomeration of Al clusters was observed throughout the physically solvent-mixed Fe_2_O_3_ nanotubes-Al nanoparticles sample (Figure 2g,h). S. H. Kim et al. reported nano-/micro-Al/Fe_2_O_3_ EMs using a simple ultrasonic mixing method, and successfully demonstrated that the Al/Fe_2_O_3_ EMs can act as an effective heat energy source for melting the SAC 305 (Sn: 96.5 wt%, Ag: 3.0 wt% and Cu: 0.5 wt%) powder layer and as a bonding medium to realize the alloying reaction of two metal (Al/Cu) substrates. The mechanical bonding strength of the bonded Al/Cu substrates can be controlled by adjusting the fuel-to-oxidizer ratio in the EM layer [49].

**Figure 3:**
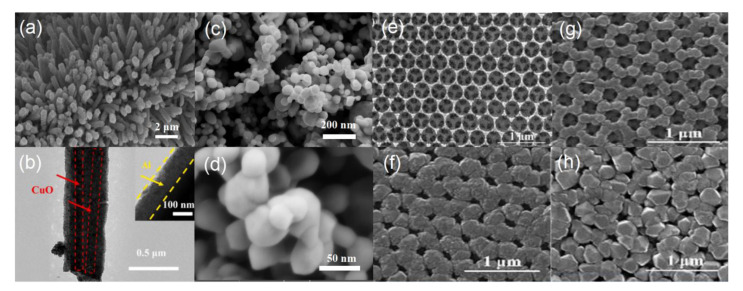
Typical FESEM image (**a**,**b**) of Al/CuO EMs by electrochemical and magnetron sputtering method [22] Copyright 2021, Journal of Alloys and Compounds, typical FESEM images with low (**c**) and high (**d**) resolution of the superhydrophobic Al/CuO energetic films [54] Copyright 2018, Materials Letters, (**e**) the 3D porous CuO prepared bycolloidal crystal template of polystyrene microspheres [56], followed by (**f**) the 3D Al/CuO EMs after magnetron sputtering of Al with different thicknesses of (**f**) 200 nm, (**g**) 100 nm, and (**h**) 300 nm, respectively. Copyright 2020, Chemical Engineering Journal.

In comparison, another typical energetic system, Al/CuO EMs, has been also intensively investigated because of its large energy density, high theoretical heat (~4.08 kJ/g) of reaction, and high reactivity [8,23,37,50,51]. Due to the uniqueness of nature of CuO, it can be synthesized into different structures, including rods, wires, tubes, porous, or sphere structures. Thus, Al/CuO EMs with more and more novel structures have been explored, showing promising ignition and combustion performance. For the core-shell CuO/Al EMs in Yang et al. [22] and reports, CuO nanowires as core are fabricated by electrochemical methods; subsequently, Al as shell is sputtered on CuO nanowires (Figure 3a,b), indicating an effective technique, which is also demonstrated by C.P. Yu et al. [52] and X Zhou et al. [53]. The target CuO/Al EMs showed a great heat-release process [22,52,53], a low reduction of activation energy (181.142 kJ/mol) and a lower ignition energy of only 10 mJ [22]. The superhydrophobic Al/CuO film was fabricated by electrophoretic deposition using the mixture of ethanol and acetyl acetone as the optimal dispersant. The target energetic film shows good structural superiority (e.g., even distribution, porous, nanoscale-Al or CuO, as seen in Figure 3c,d) and stable heat-release capacity for at least one year [54]. In addition, Al/CuO EMs with different microstructures, including the novel leaf or flaky-like CuO/Al EMs, were designed by hydrothermal reaction and mechanical mixing [55], and the 3D-ordered macroporous Al/CuO EMs were also fabricated by colloidal crystal template of polystyrene microspheres combined with magnetron sputtering technique, and the target film showed a three-dimensional and macroscopically ordered network with not only inter-connected, but also controllable distributed porosity (Figure 3e–h), possessing higher heat release (2541.4 J/g for CuO/Al with the deposition thickness of 200 nm) compared to that of the mechanically mixed sample, and a low activation energy (213.36 kJ/mol) and high combustion pressure/pressurization rate (24.6 MPa/647.98 GPa/s), respectively [56]. Another Al/CuO EM with even, multilayer structures were neatly obtained by the magnetron sputtering method [57]. In addition to exploring different structures, research into thermite reactions or the definition mechanisms of Al/CuO EMs are also receiving increasing attention. For example, C. Rossi group [58] reported a new 2D nonstationary model implementing both oxygen and Al diffusion and solving the differential equations for heat and mass transport and chemical reactions, and reached interesting results regarding the inverse evolution of flame front width with respect to the reaction front velocity, contributing to boosting the reaction velocity and the upfront heating after adding a metallic particle into the Al/CuO energetic system due to the high thermal conductivity of added metal. Additionally, in Al/CuO EMs, the nature of the monolayer interface between CuO and alumina/Al is the key factor in controlling the kinetics of Al diffusion, providing a theoretical reference for understanding the essence of the thermite interfacial reaction [59].

Moreover, there are several other representative oxidizers such as Co_3_O_4_, NiO, MnO_2_, MoO_3_, WO_3_, and Bi_2_O_3_ also used to design Al/metal oxide EMs. Al/Co_3_O_4_ EMs can show high output of heat (~9.6 kJ/g) and a mild detonation pressure (12.6 ± 1 to 20 ± 2 MPa) [60], and highly exothermic super-hydrophobic Al/Co_3_O_4_ EMs have been prepared by an electrophoretic assembly and surface energy treatment method [15,61]. The target energetic films show nanoscale and even mixing, and a violent deflagration process after capacitive detonation ignition, a low activation energy, and superlong hydrophobic stability, underscoring the significance of the exothermic stability in long-term humid environments. The Al/NiO EMs usually show a great advantage in the design of a low-gas trigger or igniter due to the lesser amount of gas produced during the thermitic reaction of Al and NiO. The theoretical gas production was ca. 2% of that from Al/CuO EMs [62]. As a comparison, the heat-release property of Al/MoO_3_ EMs was also explored due to the high theoretical stoichiometric thermite reaction of 4703 J/g [63]. Moreover, among the common EMs, the Al/Bi_2_O_3_ EMs show ultra-high deflagration and shock pressure, which are widely used as a gas sensors. For example, compared with Al/CuO, Al/MoO_3_, and Al/PTFE EMs, the Al/Bi_2_O_3_ with highest unconfined burning rate of 420 ms shows the maximum pressurization rate (~5762 kPa/μs) and shortest delay time (5 μs) to reach the maximal pressure, better than the others: 172, 35, and 33 kPa μs^−1^, and 15, 110, and 550 μs for Al/CuO, Al/MoO_3_, and Al/PTFE, respectively [64].

#### 2.1.2. Al/Metal tEMs

**Figure 4:**
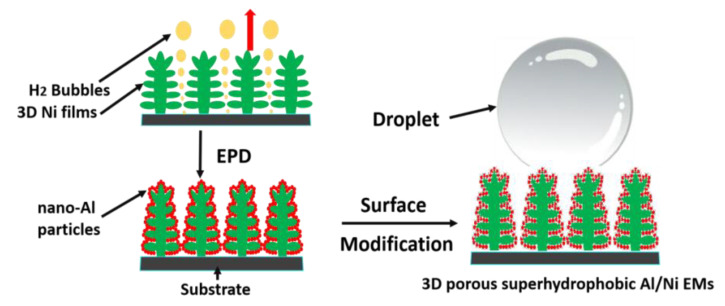
The schematic diagram of the synthesis process for 3D porous superhydrophobic Al/Ni EMs [69]. Copyright 2016, Applied Surface Science.

As a relatively special branch of tEMs, Al/metal EMs, which contains different metals, can release immense energy due to the intermetallic reaction process, and can also generate high reaction temperature, showing great potential application value, especially in welding and alloy forming. Because of the growing enthusiasm for research, this kind of Al/metal EMs has high performance requirements, so there are relatively few research objects to choose from. Current studies mainly focus on Al/Ti, Al/Ni, and so on. Thompson et al. researched synthesized multilayer Al/Ni/composite films by electron beam alternating evaporation method, and the burning rate could be as high as 400 m/s after electric heating excitation [65]. M. Milosavljevic group prepared nano-scale Al/Ti multilayer composite films by magnetron sputtering [66]. Moreover, Wang Liang’s research group studied the exothermic reaction processes of Al/Ti and Al/Ni using numerical simulation method, compared with the experimental data, which provides a favorable reference for the research of other metal composite energetic materials [67]. It is worth mentioning that in our group, the Al/Ni nano-composite energetic film was designed by an electrophoretic deposition method after optimizing suspension composition [16], showing great deflagration with a heat output of 316.2 J/g, and its exothermic stability was greatly improved after fluoride surface modification treatment [68]. In addition, promising 3D porous superhydrophobic Al/Ni EMs with great application value have been fabricated via a simple two-step method combined with hydrogen bubble dynamic template and electrophoretic deposition technique after 1H, 1H, 2H, 2H-perfluorodecyltriethoxysilane treatment [69], and the schematic diagram of the synthesis procedure is displayed in Figure 4, providing a promising novel technique for designing micro/nano energy materials for longer-term storage or transportation, especially in high humidity environments.

#### 2.1.3. Al/Organic or Macromolecule Compound tEMs

**Figure 5:**
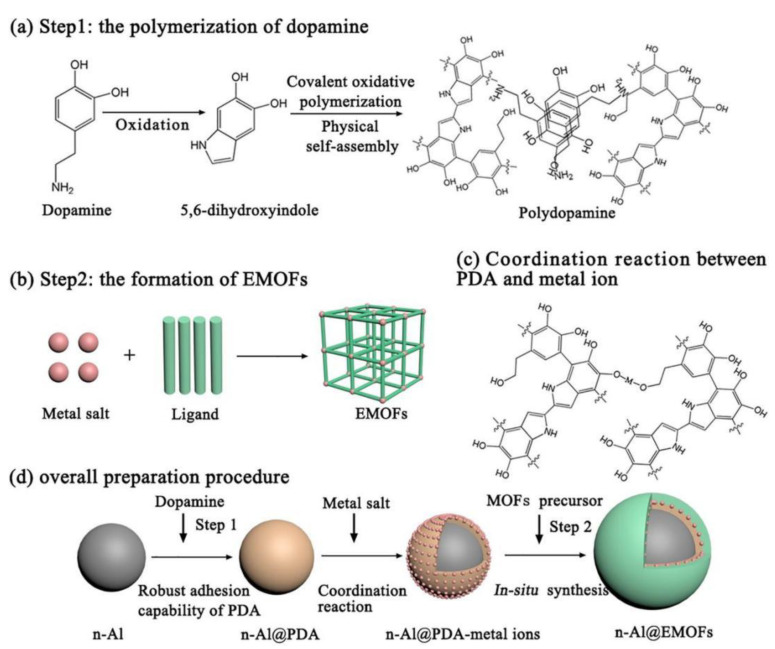
Schematic illustrations of the core-shell n-Al@EMOFs tEMs: (**a**) the polymerization of dopamine; (**b**) the formation of EMOFs; (**c**) metal-chelating reactions between PDA and metal ions; (**d**) the overall preparation procedure [75]. Copyright 2020, Chemical Engineering Journal.

As a new style of tEMs that have widely been reported on at home and abroad in recent years, Al/organic or macromolecule compound tEMs have attracted much interest recently. The appropriate organic or macromolecule compounds should have strong oxidation property, usually including Teflon or polytetrafluoroethylene (PTFE), perfluoropolyether (PFPE), trinitrotoluene (TNT), hexanitrohexaazaisowurtzitane (CL-20), metal-organic frame (MOF), and other high-energy nitramines. For example, the reaction kinetics of Al and polytetrafluoroethylene (PTFE or Teflon) were recently analyzed using nanoparticles of both Al and Teflon [70], and the nanoparticles with smaller particle size were more beneficial to the unique pre-ignition reaction, delaying the decomposition temperature of teflon and facilitating the Al/Teflon reaction. Compared with the nano-Al/R_X_O_Y_ (R = Bi, Cu, Mo) EMs, the micron-sized PTFE/Al showed the lowest burning and pressurization rate and longest delay time to reach the maximal pressure [64]. Moreover, using a situ-synthesized polydopamine (PDA) binding layer can effectively improve energy release and reduce sensitivity and, more importantly, tunable reactivity in an integrated n-Al@PDA/PTFE EMs, compared with that of the traditional n-Al/PTFE EMs [71]. Moreover, N. A. Clayton reported on Al/PFPE EMs based on polystyrene fibers via an electrospinning technique, and found that the increased loading of n-Al/PFPE results in a decrease in temperature of decomposition, and the loading of the energetic blend into the fibers showed little effect on the combustion rate from flame propagation experiments [72]. Brousseau group [73] found that compared with a micron-sized Al/TNT mixture, the use of nano-Al can reduce the critical diameter of the mixture and increase the combustion and heat release. In addition, an interesting “father-son” exothermic effect in the promising Al/[Mn(BTO)(H_2_O)_2_]n (BTO = 1H,1′H-[5,5′-bitetrazole]-1,1′-bis(olate)) prepared through a simple ultrasonic dispersion method was due to the contribution of the “father” [Mn(BTO)(H_2_O)_2_]n’ thermal decomposition reaction and the “son”-metal oxide’s thermite reaction with nano-Al, showing the excellent energetic properties [74]. A novel and promising energy-storage system using energetic metal-organic frameworks (EMOFs) as the oxidizers has been proposed recently [75], and the preparation mechanism is shown in detail in Figure 5. The energetic MOF-activated Al (n-Al@EMOFs) with the multilayer core-shell structure guarding against n-Al oxidation displays the unique two-step exothermic processes (n-Al@EMOF→n-Al@PDA + CuO + Others and n-Al@PDA + CuO→Al_2_O_3_ + Cu + others) with a total heat release of ~4142 J/g), and lowers the ignition temperature to 301.5 °C, which lays the groundwork for the development of novel tEMs.

#### 2.1.4. Al/Inorganic Compound tEMs

Inorganic compounds are regarded as another attractive oxidizing agent to be applied to tEMs due to their low-cost and convenient preparation process, and the reported inorganic salts include KMnO_4_, Na/KIO_4_, NaClO_4_, etc. For example, Zachariah et al. [76] reported on Al/KMnO_4_ EMs using KMnO_4_ as the oxidizing agent due to its high volatility, strong oxidation nature, and low decomposition temperature (~300 °C), and the target EMs showed ultra-fast reactivity and a pressurization rate of 290 psi/μs, compared with that of Al/Fe_2_O_3_, Al/CuO, and Al/MoO_3_. Moreover, the same group also designed Al/periodate salts (NaIO_4_ and KIO_4_) EMs [77], whose maximal detonation pressure can reach ~40 atmospheres within a very short time (~0.01 ms). The direct gas phase oxygen release from the NaIO_4_ and KIO_4_ decomposition is key to the ignition and combustion of Al/periodate EMs, showing a great advantage in designing super-reactive nano energetic-based gas generators. AgIO_3_ was selected as a special oxidizer for systems designed for biocidal activity. The Al/AgIO_3_ EMs have multiple advantages, including a faster deflagration process, better pressurization enhancement ability due to the released gases (O_2_ and I_2_) from AgIO_3_ than the traditional Al/CuO and Al/Fe_2_O_3_ EMs, and a long-lasting biocidal nature, indicating a wide range of potential thermite-based biocidal applications [78].

### 2.2. Ternary tEMs

With the increasing improvement requirements for performance, including increasing output of heat, and adjusting the reaction velocity, energy density, or sensitivity, ternary tEMs with single or multiple fuels and oxidants have gradually entered the field of vision of researchers. For example, ammonium perchlorate (AP), as a widely used oxidizing agent in solid rocket propellant, has been added in Al/Fe_2_O_3_ EMs to obtain AP/Al/Fe_2_O_3_ ternary nano-thermites [79], showing a lower activation energy of 109.22 kJ/mol and a stronger exothermic peak, with the output of heat of ~1.82 kJ/g, which is 1.3 times that of the simply mixed sample. Several other additives of polytetrafluoroethylene (PTFE) [80], graphene oxide [81], hexogen (RDX) [82] and SiO_2_ [83] have been added into traditional binary Al-based EMs, forming the corresponding ternary tEMs of Al/Bi_2_O_3_/PTFE, Al/CuO/graphene oxide, Al/Fe_2_O_3_/RDX and Al/Fe_2_O_3_/SiO_2_, respectively. The addition of nonmetals (boron, etc.) or (Ni, etc.) can also enhance the exothermic capacity of tEMs. For example, thermodynamically, boron (B) releases more energy on both a mass and volumetric basis, thus, nano-B can enhance the reactivity of Al/CuO EMs when added as the minor component (<50% by mole) of the fuel [84]. The ternary composite Al_x_Ni_y_(Bi_2_O_3_)_z_ has also been designed and showed better heat release than that of Al/Ni EMs [16] and Al/Bi_2_O_3_ EMs, also processing excellent thermal stability property at least for nearly one year [85].

### 2.3. tMs with Multiple Components

**Figure 6:**
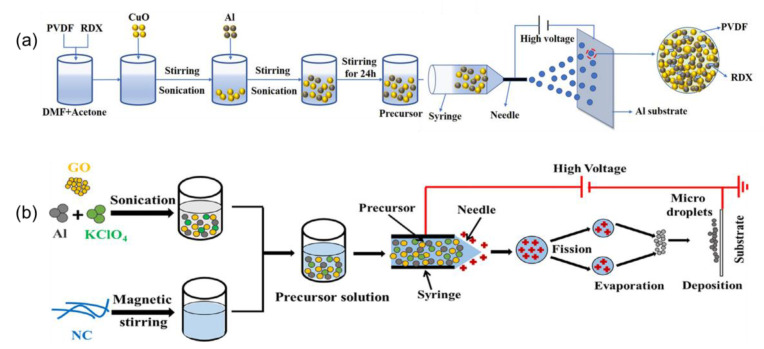
Schematic illustration of (**a**) quaternary Al/CuO/PVDF/RDX microspheres [86] Copyright 2021, Chemical Engineering Science, and (**b**) quaternary NC/GO/Al/KClO_4_ nEMs [87], respectively. Copyright 2021, Combustion and Flame.

Additionally, tEMs with multiple components comprise a promising branch of energetic materials. At present, there are also a few reports on adding multiple additives to design novel tEMs. For instance, the Al/CuO/(polyvinylidene fluoride) PVDF/RDX energetic microspheres assembled by an electrospray technology method (Figure 6a) display a shorter delay time, burning time, and higher pressure, pressurization rate than the physical mixtures, and the content of RDX effectively regulates the combustion performance of the product [86]. A. Fahd et al. reported on the quaternary nitrocellulose/GO/Al/KClO_4_ EMs (Figure 6b) using a facile electrospinning method. The addition of nitrocellulose (NC) not only contributes to improving the heat-release process, but also introduces another step to the GO/Al/KClO_4_ reaction of a gas–solid phase and liquid–liquid phase diffusion reaction, with the liquid–liquid phase reaction increasing with the content of NC [87]. Adding different inert materials into tEMs will be an effective strategy to solve the safety issue, and adjust exothermic performance and sensitivity according to specific requirements.

## 3. Synthesis Methods for tEMs

### 3.1. Mechanical Mixing Method

The mechanical mixing method is the most common and widely used technique to obtain tEMs with an appropriate reaction measurement ratio after accurate weighing. The method does not need large-scale instruments, is simple to operate, and has a wide application range. Thus, lots of researchers at home and abroad use this approach to study the properties of tEMs. For example, as one of the most frequently studied systems, the Al/CuO tEMs is conveniently prepared by the ultrasonic mixing method [55,88]. The K. Lee group proposed that the maximum pressurization rate occurs in Al-rich thermite composites due to the higher deposition rate of Cu on the surface of Al particles during thermite reaction than that of the whole heat-release reaction, rendering Al NPs relatively less reactive too the activity [88], which provides valuable reference for designing other tEMs with better exothermic performance. The heat release performance of four systems: Al/MoO_3_, Al/CuO, Al/Bi_2_O_3_, and Al/WO_3_ EMs, were systematically analyzed by the mechanical mixing method [89], and the relationships between the molar ratio of different components and heat release, explosion pressure, and burning rate were explored, respectively. In addition, K.S. Martirosyan et al. [37] investigated eight kinds of EMs: Al/Fe_2_O_3_, Al/Fe_3_O_4,_ Al/MoO_3_, Al/MoO_2_, Al/CuO, Al/WO_3_, Al/Bi_2_O_3_, and Al/MnO_2_, and concluded that the explosive pressure of Al/Bi_2_O_3_ EMs was the largest, producing a ultra-high pressure pulse of nearly 100 atmospheres, and the peak pressure generally increases using smaller Al and/or bismuth oxide particles, which proved that Al/Bi_2_O_3_ tEMs has a great application prospect in gas sensors. Undeniably, the mechanical mixing method is a simple and effective method for screening valuable or superior tEMs, but it shows no advantage for film-forming combined with different devices used in various applications.

### 3.2. Vapor Deposition Method

Vapor deposition method is regarded as an effective technique for designing tEMs with controllable stratified structures. The key of this method is how to change solid or liquid material into gas by means of external energy, including electromagnetic energy, thermal energy, etc., and to control it precisely.

**Figure 7:**
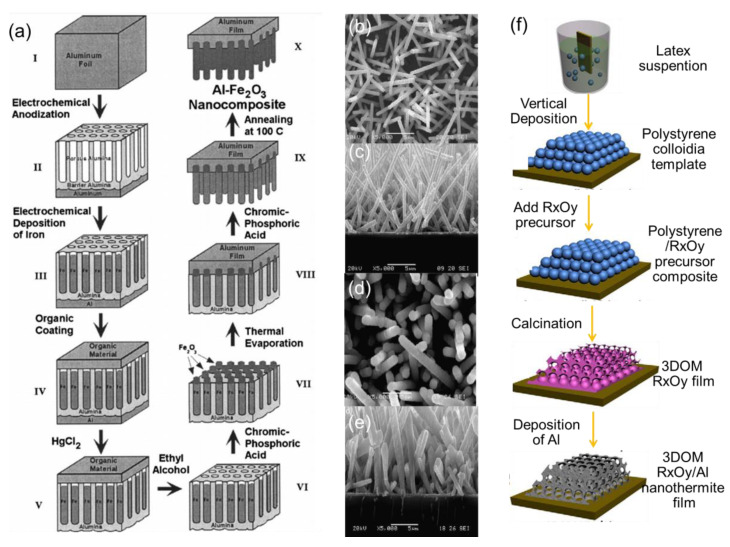
(**a**) The steps for preparing Al/Fe_2_O_3_ energetic nanocomposites [94] Copyright 2012, Journal of Applied Physics, SEM images ((**b**) top view and (**c**) cross-section view) of the Co_3_O_4_ nanorods; SEM images ((**d**) top view and (**e**) cross-section view) of the Al/Co_3_O_4_ nEMs [96] Copyright 2012, Combustion and Flame, and (**f**) the schematic of the synthesis procedure for 3D ordered macroporous (3DOM) R_X_O_Y_ nanothermite films, (R = Fe, Co and Ni) [98] Copyright 2016, Materials and Design.

One of the most common and effective methods is magnetron sputtering, which is a method of using high-speed charged ions accelerated by the external electric field to collide with the target sputtering material, and under the control of certain appropriate parameters such as voltage, frequency, electromagnetic field, etc. The main advantage of the magnetron sputtering technique is the high degree of controllable thickness of a single layer among layered tEMs. For example, multi-layer Al/Ni tMEs with different layer thicknesses 50–200 nm were prepared by the magnetron sputtering method [90], and their exothermic performances were largely affected by the layer thickness. The highest electrical explosion temperature of 7000 K occurred when the layer thickness was 50 nm. Until now, lots of frequently reported tEMs, such as Al/Ti [66], Al/MoOx [91], and Al/CuO [92], have been designed by this method, applied in trigger and detonator with superior performances. Another common method is using thermal evaporation to fabricate tEMs with outstanding properties. For example, Al/NiO EMs based on silicon substrates have been fabricated by the thermal evaporation technique, showing the advantages of enhanced interfacial contact area, lowered ignition temperature (~400 °C), reduced impurities, tailored dimensions, and strong heat capacity (~2.2 kJ/g) [93]. The sequence of steps to prepare Al/Fe_2_O_3_ EMs via this method (Figure 7a) was proposed by L. Menon et al. [94]. Moreover, atomic-layer deposition is another approach using uninterrupted, sufficient gas-solid reactions to obtain target coatings. For example, Al/ZnO EMs with stratified or core-shell structures [95] have been successfully prepared by using the atomic-layer deposition technique, and the reaction rate of the core-shell-structured Al/ZnO EMs was several times faster than that of the simple mixed sample under laser ignition experiments.

In addition, several recent reports have focused on a combination method of vapor deposition and other techniques to design novel tEMs. For example, the Al/Co_3_O_4_ EMs were also successfully fabricated by chemically synthesizing Co_3_O_4_ nanorods as the core (Figure 7b,c) and depositing Al layers as the shell (Figure 7d,e) using thermal evaporation, and the total heat of reaction was as high as 3635 J/g, benefiting from the full contact core-shell structure [96]. The Al/NiO or Fe_2_O_3_ or Co_3_O_4_ EMs [97,98,99] with a novel ordered macroporous structure was prepared by polystyrene colloidal template technique and thermal evaporation or magnetron sputtering method, and the corresponding schematic of the synthesis procedure is displayed in Figure 7f.

### 3.3. Assembly Methods

#### 3.3.1. Electrophoretic Assembly Method

**Figure 8:**
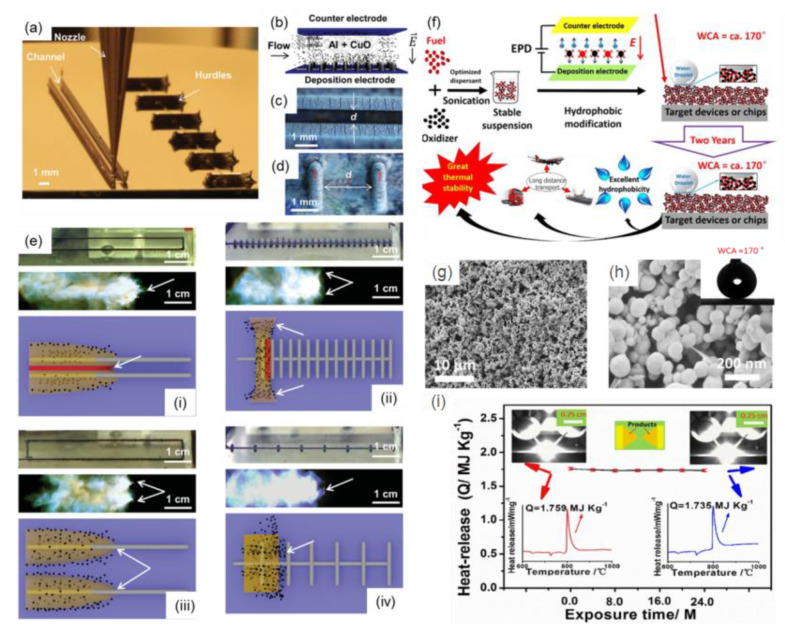
(**a**) Optical image of 3D printing process for channels (left) and hurdles (right) composed of silver nanoparticle ink, (**b**) schematic illustration of the electrophoretic assembly process of Al/CuO EMs onto the electrode surfaces, (**c**) optical images (top view) of a channel, (**d**) hurdle architectures after deposition of Al/CuO film, (**e**) optical image of four kinds of channel and hurdle structures, followed by the corresponding combustion process and the resultant pressurization region and expansion process illustration [104] Copyright 2016, Advanced Materials; (**f**) the schematic diagram of the fabrication of superhydrophobic Al/Bi_2_O_3_ films, the SEM images ((**g**) low resolution and (**h**) high resolution) of product, and (**i**) the thermal stability property of product after different exposure time. Inset, DSC and ignition tests reveal the exothermic performance of products before and after two years exposure [111], Copyright 2018, Chemical Engineering Journal.

The electrophoretic assembly method is regarded as a convenient and low-cost technique to assemble differently charged micro/nanoparticles or polymer molecules to form even target films on substrates with different structures [100,101,102], including tiny devices used in micro-electro-mechanical systems (MEMS). Recently, different kinds of tEMs with controllable structures and properties were successfully fabricated by the electrophoretic assembly method. For example, Al/CuO binary tEMs were obtained by the electrophoretic assembly technique on a simple two-dimensional surface [103] and complex 3D printing substrate with channels and hurdles (Figure 8a) [104]. The schematic illustration of the electrophoretic assembly process is shown in Figure 8b, followed by optical images of channel (Figure 8c) and hurdle architectures (Figure 8d) after deposition of the target energetic films. The tiny energetic devices with differently designed electrode spacing and direction distributions showed different orientations of the multiphase expansion event relative to the flame propagation direction (Figure 8e). The electrophoretic assembly film-forming of other common tEMs (e.g., Al/NiO [62], Al/Ni [16], Al/Fe_2_O_3_ [105], Al/Co_3_O_4_ [106], Al/MoO_3_ [107], Al/WO_3_ [108], etc.) have been also demonstrated, especially in the last decade.

Several tEMs with promising structures and stable exothermic performance can be prepared by combining electrophoretic assembly and other fabrication methods. In order to solve the problem of poor exothermic stability of energetic materials due to the hydrophilicity of Al-based materials, anti-wetting EMs (e.g., Al/Fe_2_O_3_ [109], Al/ZnO [110], Al/Ni/Bi_2_O_3_ [85], Al/Bi_2_O_3_ [111], Al/MoO_3_ [112], Al/NiO [113], Al/Ni [69], Al/Co_3_O_4_ [61], Al/CuO [54]) have been designed by electrophoretic assembly and surface modification process. For example, Guo et al. [111] reported on superhydrophobic Al/Bi_2_O_3_ films using the mentioned approach by per-fluoroalkyltriethoxysilane (FAS-17) modification, and the preparation mechanism is shown in Figure 8f. The product is rough but evenly distributed in nanoscale (Figure 8g,h), which improves combustion performance significantly. The superhydrophobic Al/Bi_2_O_3_ films show an outstanding anti-wetting property, with a water contact angle of ~170° and high exothermic stability for 2 years (Figure 8i).

#### 3.3.2. Other Assembly Method

Recently, some novel promising assembly methods have also been explored to improve the contact area and reactive activation of tEMs. The main driving forces for assembly methods include the electrostatic force, strong affinity of the functional groups, biomolecular driving force, and so on. Electrostatically enhanced nano-Al and nano-Fe_2_O_3_ particle self-assembly was realized to design reactive Al/Fe_2_O_3_ tEMs, and the burning process of the resulting product is controlled by adjusting the magnitude of the particle charge [114]. The Al/NiO tEMs are fabricated via co-assembly with poly(4-vinylpyridine) (P4VP), and the structure of the Al/NiO nanocomposites with P4VP is more regular and compact, resulting in a higher output of heat (2190 J/g), a higher maximum explosion pressure (0.35 MPa), a faster pressure rise rate (260 MPa/s) and burning rate (462 m/s), compared with those of a physically mixed sample [115]. In addition, C. Rossi et al. [116] reported a novel technique of DNA (e.g., a linker with sequence 5′ to 3′ of GAGGGATTATTGTTAd)TTAACGTACAGTATG)-directed assembly procedure to obtain highly Al/CuO tEMs, showing a total highest heat of reaction of 1800 J/g for the 80 nm Al NPs, and the onset temperature can be adjusted by changing the size of Al particles.

### 3.4. Sol-Gel Method

The sol-gel method is a widely used method to prepare nanometer metals, oxides, or metal/oxides. To be specific, the selected raw materials are dispersed into a solvent as a precursor, and they then form the colloidal solution (sol) with high dispersion after hydrolysis, and certain space structures (gel) are finally obtained after the polymerization process with other components added. Tillotson et al. prepared the aerogel and xerogel monoliths of Al/Fe_2_O_3_ tEMs using the sol-gel method, all releasing dazzling white light after ignition, and the aerogel composites were more sensitive to ignition than that of the aerogel sample [117]. In addition, a ternary tEMs of Al/Fe_2_O_3_/SiO_2_ thermite was designed by this method, and examining the influence of the mass fraction of SiO_2_ as an additive display showed a great effect on the exothermic performance and combustion velocity [118]. The sol-gel method is simple and can be operated in a beaker. However, there are many impurities in the product that will greatly affect the performance of the target product. In addition, similar to the high-energy ball milling technique, the microstructure of the product is difficult to control, thus, the general applicability of this method is still limited.

### 3.5. Electrospinning Method

**Figure 9:**
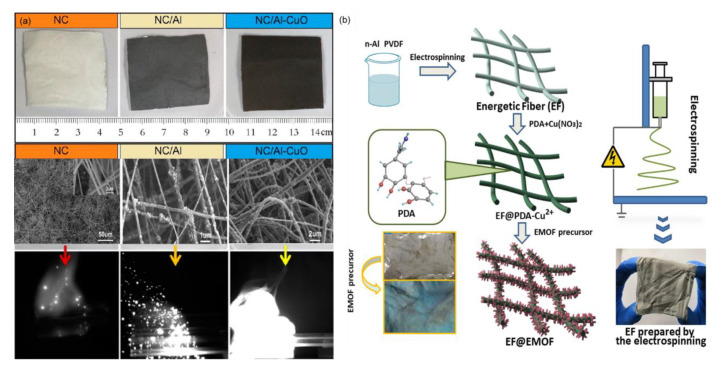
(**a**) The optical and SEM image of pure NC, NC/Al (50 wt%) and NC/Al-CuO (50 wt%), followed by the respective burning static snapshot [120] Copyright 2012, ACS Applied Materials Interfaces, and (**b**) the general procedures for the preparation of n-Al@PVDF/EMOF (DHBT) [121] Copyright 2020, Chemical Engineering Journal.

As a facile and highly versatile technique, electrospinning is also used to generate multifunctional ultrathin fibers from various polymers, polymer blends, and polymer nanoparticles [87]. For example, nitrocellulose (NC) as the chief ingredient in single-base and double-base gun and rocket propellants has been introduced into Al/Fe_2_O_3_ tEMs to obtain Al/Fe_2_O_3_/NC energetic fibers by an electrospinning method [119], and the product with the increased elastic modulus of NC fibers after the addition of 5 wt% Al/Fe_2_O_3_ shows a lower onset temperature of thermite reaction compared to Al/Fe_2_O_3_/NC powders. Another similar report is the design of Al/CuO/NC tEMs by M. R. Zachariah et al. The color and morphology of the samples changed obviously compared with pure NC, NC/Al, and Al/CuO/NC tEMs, and regarding the Al/CuO being electrospun in the NC polymer matrix, the deflagration process is more complete and the flame area is larger and more dazzling (Figure 9a), showing wide applications in solid rocket propellant systems [120]. Promising n-Al@PVDF/EMOF (DHBT) highly energetic fibers were fabricated by Q.L. Yan group using the electrospinning method combined with a typical in situ synthesis process (Figure 9b), and the product shows increased heat release (3.464 kJ/g) and burning rate (2.8 m/s), and improved combustion efficiency due to the greater amount of reaction channels after the decomposition of EMOF and the etching reaction [121].

### 3.6. Other Methods

In addition to the method mainly used for designing tEMs, researchers at home and abroad tried to explore other preparation technologies, including arrested reactive milling [122,123], reactive plasma spraying [124], electrospray deposition [125], powder metallurgy [126], the freeze-drying method [127], the hydrothermal synthesis method [128] and the brush-mixing technique [129]. Interestingly, J.M. Slocik et al. constructed energetic biothermite inks, that is, nAl@ferritin liquid tEMs using the freeze-drying technique, and demonstrated greater processability and functionality, increased energy output and performance, enhanced dispersion and oxidation stability, and lower activation temperatures (<400 °C) [127], which provides valuable reference for designing Al-based energetic biological liquids with flexible structures and shapes using different biological activities or other various capabilities.

## 4. Prospects and Suggestions

Until now, energetic materials, particularly tEMs, have been extensively reported and fabricated via different preparation methods, owing to their wide range of application, which include the defense industry, detonators [128,129,130,131], igniters [132,133], gas sensors [77,134,135,136,137], thin-film battery [138] and chip adhesive [139]. Beyond that, the design of an all-solid-state lithium/electrolyte interface was realized by inducing an energetic reaction between metal fuel and nitrate, showing high capacity, small overpotential, high efficiency, and a long cycle life [140]. Bacteria growth neutralized by Al/I_2_O_5_ tEMs with great biocidal property was demonstrated by B. R. Clark et al. [141]. The means by which energetic materials can be boldly and organically combined with other fields will be a hot topic that can be continuously paid attention to in the future.

There is no denying that more and more techniques are being developed for designing tEMs, including those mentioned in this review, such as the mechanical mixing method, vapor deposition methods, assembly method, sol-gel method, electrospinning method, freeze-drying method, etc. However, as the application of tEMs in the fields (e.g., national defense) becomes more and more demanding, new requirements are put forward for the development and exploration of novel technologies. For example, although the mechanical mixing method is simple and convenient, it does not allow for film formation and integration with complex micro-devices. Although they have high precision and controllability, it is still difficult for them to achieve large-scale production due to the high preparation cost and difficult operation of magnetron sputtering, electrospinning, etc. The sol-gel method has great advantages in preparing porous tEMs with good contact, but the purity of the product often requires subsequent impurity removal processes, such as heat treatment, that have increased limitations for tEMs with high thermal sensitivity, though it is relatively efficient. For arrested reactive milling, its preparation cost is low, but the size of the molding sample is mostly micron, leading to a limited heat-release performance. Moreover, the low loading rate and adhesion to the substrate using the electrophoretic assembly method needs to be further improved, though it is rather convenient and has high film-forming efficiency, being perfectly matched with devices (e.g., chips). The generality and universality of new technologies (e.g., freeze-drying method) for tEMs need further verification. Therefore, in the future, the means by which novel methods with high universality, high safety of preparation process, environmental friendliness, and a better combination of reported mature traditional technologies can be developed should be the focus of additional research.

Exothermic performance optimization is an essential index to evaluate the application of tEMs. The key to thermal energy release is the design of microstructures, which mainly are stratified, nucleated, or granular. It is rather urgent and necessary to design novel structures to further shorten the mass transfer distance between fuel and oxidizer, improve the heat transfer efficiency, and meet the requirements of ignition and heat release under complex conditions, such as acid, alkali, wet environments, underwater environments, etc.

**Figure 10:**
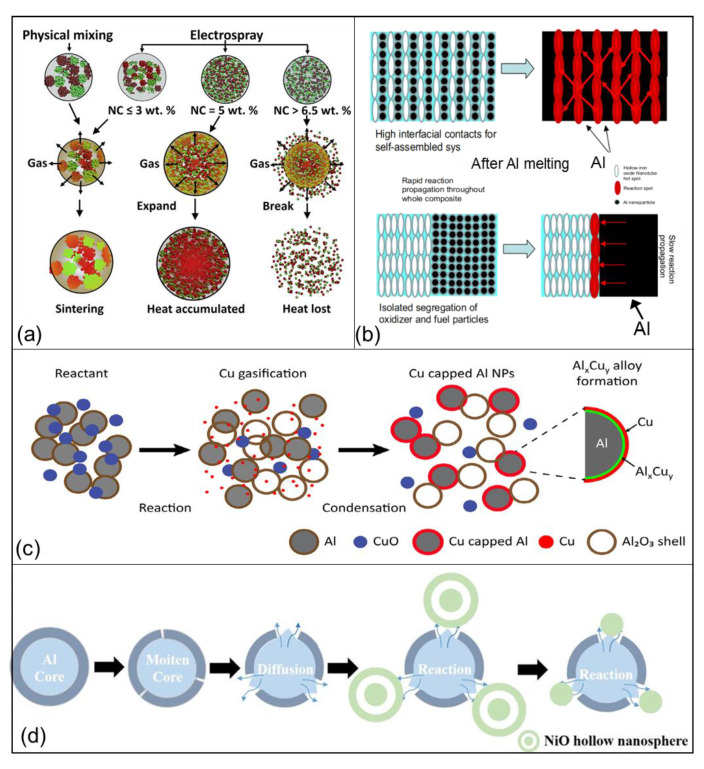
(**a**) Possible mechanisms of the burning of Al/CuO/NC nEMs made from physical mixing and electrospray. Note: Al (red); CuO (green); nitrocellulose (light blue) [142] Copyright 2014, Combustion and Flame, (**b**) the schematic illustration showing an exaggerated perspective that demonstrates random versus ordered assembly [48] Copyright 2010, Combustion and Flame, (**c**) schematic illustration of Al/CuO thermite reaction steps. Condensation of gasified Cu species on Al NPs hamper’s reaction of Al NPs with neighboring CuO NPs [88] Copyright 2015, Combustion and Flame, and (**d**) combustion process of Al/NiO nEMs [131] Copyright 2020, Chemical Engineering Journal.

Additionally, the analysis of mechanism is a long-term research focus, which plays a significant role in optimizing the heat-release property of tEMs. The main research has focused on the improvement of their performance and structure optimization. Admittedly, several researchers have explored the reaction mechanism of tEMs. For example, M. R. Zachariah proposed possible mechanisms of the burning process of Al/CuO/NC nEMs fabricated by two different physical mixing and electrospray techniques (Figure 10a), and found that adding additional NC (>6.5 wt%,) apparently offers diminishing returns, leading to its disintegration into smaller structures and its pressure and the pressurization rate becoming smaller, respectively [142]. Heat-release schematic illustrations of Al/Fe_2_O_3_, Al/CuO, and Al/NiO nEMs with different microstructures (Figure 10b–d) have also been analyzed recently [48,88,131]. However, for the complex system of tEMs, the mechanism research is far from enough. Therefore, the relative mechanisms and simulation (via COSMOL, DSC, etc.) analysis of preparation, exothermic reaction and modification process, etc. also need to be focused on for tEMs, especially multi-component tEMs.

## 5. Conclusions

In brief, this review systematically introduces the classification, various synthesis methods, and recent research achievements of highly reactive tEMs with broad and promising applications. Until now, a great deal of promising work has been reported to prepare tEMs for optimizing their performance. Due to the particularity of tEMs, the development of new methods with high universality and environmental friendliness needs to be focused on in future research, even though there are many technologies reported at present. The family and application breadth of tEMs needs to be further expanded and applied in fields such as intelligence, biology, information technology, etc. Additionally, the nanostructures of tEMs need to be further designed to improve the adequacy and stability of the reaction. A combination of microstructure design and exploration of technology is used to finally achieve efficient mass production. Additionally, further study is needed to explore the relative mechanism for providing a solid foundation for optimized performance. In brief, the subject of this review belongs to a youthful and promising research area, and significant and meaningful work can be explored to expand its scope.

## Data Availability

Not applicable.
